# Synthesis and Characterization of PVDF Hollow Fiber Using Adipic Acid as an Additive for Gas–Liquid Membrane Contactor Application

**DOI:** 10.1021/acsomega.4c11065

**Published:** 2025-04-29

**Authors:** Felipe Brandão de Souza Mendes, Cristina Cardoso Pereira, Alberto Cláudio Habert, Cristiano Piacsek Borges

**Affiliations:** † Brazilian Navy Research Institute, Brazilian Navy, Ministry of Defense, 21931-095Rio de Janeiro, Brazil; ‡ PENT-COPPE, Federal University of Rio de Janeiro, 68505, 21941-972 Rio de Janeiro, Brazil; § PEQ-COPPE, Federal University of Rio de Janeiro, 68502, 21941-972 Rio de Janeiro, Brazil

## Abstract

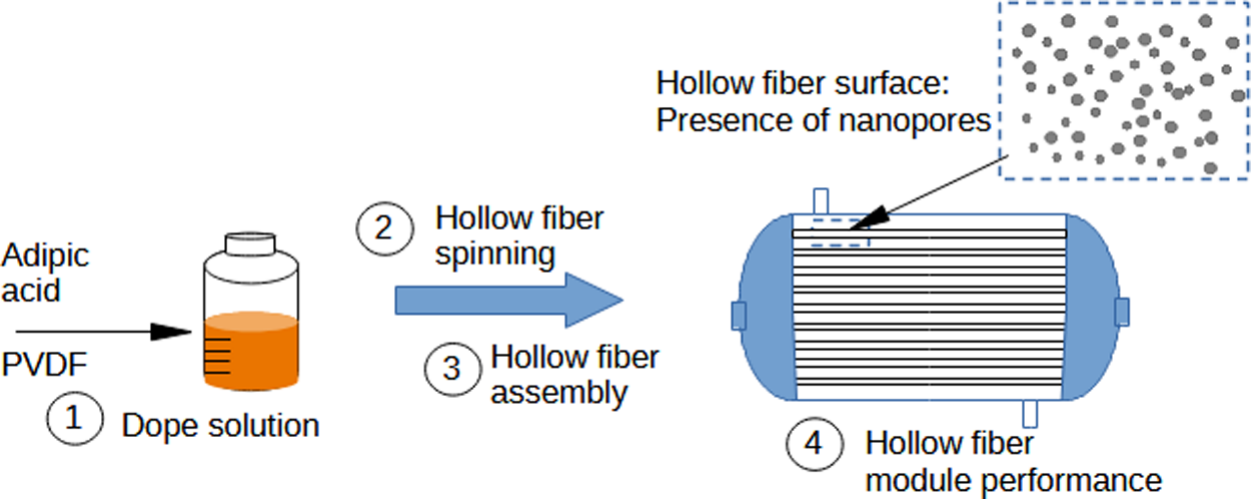

In the present work, poly(vinylidene fluoride) (PVDF) hollow fiber membranes were obtained using adipic acid as an additive in dope solution. The PVDF hollow fibers produced were used in the gas–liquid membrane contactor process, aiming at CO_2_ capture. The morphology of PVDF hollow fibers was also characterized by scanning electron microscopy and helium ion microscopy (HIM). These techniques, mainly HIM, allowed us to clearly observe the presence of nanopores at the outer membrane surface, which may favor the process efficiency by preventing membrane wetting. The hollow fiber membranes were also characterized by helium picnometry, gas permeation, and the contactor membrane process. In the performance tests for CO_2_ removal, the number of fibers and length of the PVDF hollow fibers were taken into account, since in-house modules were also compared to commercial ones. From these experiments, it could be seen that PVDF hollow fibers exhibited better performance of the CO_2_ flux than commercial polypropylene hollow fibers.

## Introduction

The removal of carbon dioxide (CO_2_) from different streams is applied to several industries. For example, CO_2_ removal is normally applied in the oil and gas industry in order to make natural gas suitable for commercialization, since CO_2_ presence reduces the gas calorific value and promotes corrosion in the pipeline. CO_2_ removal can also be applied in carbon capture from flue gas in order to avoid CO_2_ emissions into the atmosphere, as well as syngas purification.^1^ These are some of the applications aiming at CO_2_ removal that could be performed by a number of different processes, such as column absorption, pressure swing adsorption, cryogenic separation, and membrane gas permeation. Column absorption and membrane gas permeation are widely used to remove CO_2_ from natural gas. In column absorption, CO_2_ is absorbed inside the column when the liquid phase encounters the gas phase. Mass transfer is intensified due to ceramic or metallic fillers. However, some problems may occur during operation, such as flooding and channeling.^2^ Besides, scale-up of this technology is another big issue because the mass transfer area depends simultaneously on gas and liquid flow and the geometry of the filler.

**Figure 1:**
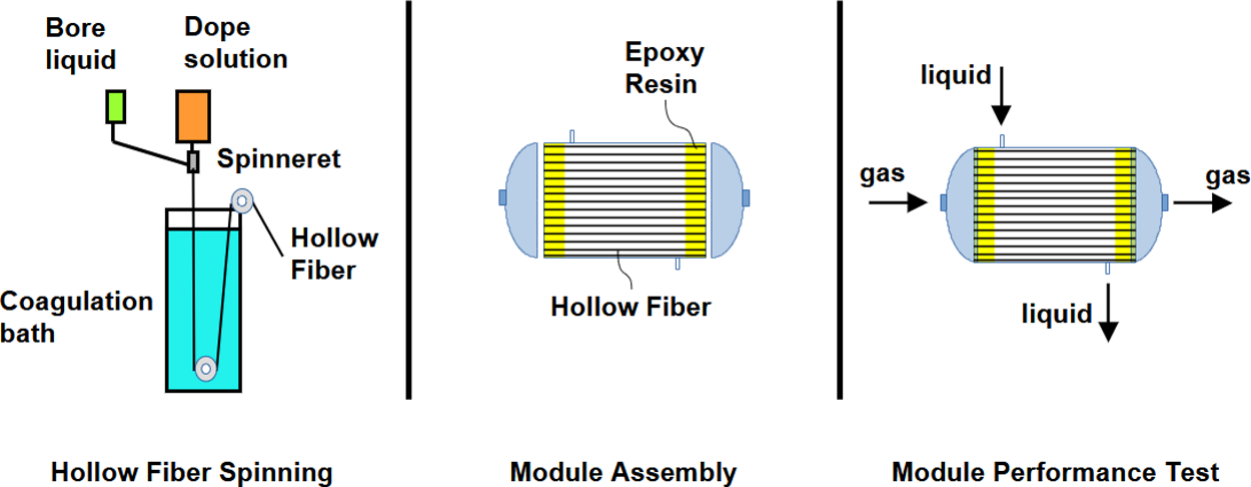
Main steps investigated in the present work, i.e., PVDF hollow fiber spinning, module assembly using the PVDF membranes produced, and its performance test.

Membrane processes have already proved to be attractive alternatives to substitute absorption columns, principally due to the reduced footprint. In fact, membrane processes for gas separation have already been used in the oil and gas industry to remove CO_2_ from natural gas. In gas separation using a dense membrane, it acts as a selective barrier that determines gas permeation due to the affinity between gaseous species and the membrane material. Due to the interaction of CO_2_ and the polymer material, one of the challenges of gas permeation is to avoid membrane plasticization. In this case, due to the penetrant dissolution in the polymer matrix, it can cause membrane swelling. This effect can increase permeability, but it drastically decreases the process selectivity.^3^ Due to these facts, membrane contactors for CO_2_ removal have been investigated for replacing conventional processes. In a membrane contactor, the membrane acts as a barrier between two phases: a gas phase with the component to be removed, such as CO_2_, and a liquid absorbent phase (depicted in Figure 1). Different from the traditional membrane processes, in the contactor process, the membrane is not selective.^4^ The selectivity depends on the liquid absorbent, which can act by physical absorption, chemical reaction, or a combination of both.^5−10^ CO_2_ in membrane contactors occurs when the gas stream comes in contact with the liquid absorbent through the membrane pores. The pores of the membrane are small enough that capillary forces predominate and inhibit the mixing of the phases present on each side of the membrane. Membrane contactors also combine the benefits of traditional membrane processes, such as a reduced footprint, a modular and compact design, and easy scale-up, and the benefits of the absorption column, which presents elevated selectivity due to liquid absorption. Besides, in the case of the membrane contactor, fluid phases can be operated independently, provided the pressure of the liquid phase does not exceed the breakthrough pressure and the interfacial area is large, known, and constant.^4,11−13^

Most commercial membranes are made up of polymeric materials. Therefore, polymers must have thermal stability in a wide range of temperatures and chemical stability at different pH values. Depending on the application, associated with these two aspects, polymers must also have high mechanical strength.^14^ Several polymers are used to produce hollow fiber membranes that can be applied in membrane contactors. Aiming at this application, the materials used to produce hollow fibers must avoid pore wetting, which reduces mass transfer, so that the performance of the process is not reduced.^15−17^ Besides the pressure difference between the gas and liquid flow, there are three aspects that can be modified, according to the Young–Laplace equation, in order to avoid wetting: the mean pore diameter at the surface, hydrophobicity, and surface tension.^18^ The last one is a characteristic of the liquid used to capture CO_2_, and consequently, it could be a process current that cannot be modified. The others are related to the membrane and can be tuned during the membrane preparation step. Therefore, clearly, the use of hydrophobic polymer materials is strongly recommended due to the use of aqueous liquid solution absorbents in the process. Polypropylene (PP) and polytetrafluoroethylene (PTFE) are the most used polymers to produce hollow fibers to membrane contactors in the CO_2_ removal process. However, some disadvantages related to these polymers can be highlighted: PTFE, despite having high hydrophobicity, has a high production cost and PP, and despite being cheaper and widely found commercially, has low chemical resistance to commonly used absorbent liquids.^2^ However, some efforts can be made to overcome these disadvantages. For example, Mulukutla et al. coated the outer surface of PP hollow fibers with a plasma polymerized hydrophobic porous fluorosiloxane, so that only the coating was exposed to the absorbent while the PP sublayer was not.^19^ Besides, PP and PTFE membranes are usually obtained by stretching or thermal methods due to their limited solubility in several solvents, promoting less flexibility for preparation. On the other hand, polyvinylidene fluoride (PVDF) is widely used in hollow fiber manufacture. Its chemical structure is formed by a repeated chain of −(C2H2F2)– and it exhibits great properties such as thermal, chemical, and mechanical stabilities.^20^

PVDF membranes can be produced by different synthesis techniques, such as thermally induced phase separation (TIPS), nonsolvent-induced phase separation (NIPS), and vapor-induced phase separation (VIPS).^21^ Ghasem et al. evaluated the effect of PVDF polymer solution concentration in pure CO_2_ flux when hollow fibers were used in membrane contactors using 0.5 M NaOH solution as the absorbent liquid.^22^ The authors concluded that the lower the polymer concentration, the higher the CO_2_ flux. They observed that polymer concentrations above 30% lead to the formation of a dense skin in the outer surface, whereas below that concentration, they observed a porous outer surface.

The use of additives in polymer solutions aims to control the hollow fiber morphology by influencing the thermodynamic and kinetic aspects during membrane formation. Additives could be salts or organic molecules with high or low molecular weight. Naim et al. investigated how the concentration of LiCl in the polymer solution affects both the morphology and the performance of PVDF hollow fibers applied in CO_2_ stripping of diethanolamine solution.^23^ The authors maintained the PVDF concentration in 17 wt % and varied the additive concentration from 0 to 5 wt %. They observed that the higher the additive concentration, the lower the mean pore diameter. Moreover, scanning electron microscopy (SEM) photomicrographs revealed that with increasing LiCl concentration, there was a reduction in finger-like pores, as well as a migration of its predominance to near the outer surface. Atchariyawut et al. prepared three polymer solutions, each one containing 17% PVDF, 80% *N*-methyl-2-pyrrolidone (80% NMP), and 3% additive, such as phosphoric acid, glycerol, and distilled water. The precipitation bath chosen was pure distilled water, and the bore liquid was a mixture of 20% distilled water and 80% NMP. This bore liquid concentration promoted the formation of a sponge-like morphology in the lumen of the hollow fiber. The authors observed a higher flux of CO_2_ for membranes prepared with distilled water, glycerol, and phosphoric acid, respectively. They attributed the increase in CO_2_ flux to the sponge-like morphology in the inner part of the hollow fiber, so that the mass transfer resistance in the gas phase decreases. Pereira et al. investigated PVDF membrane formation by NIPS using two different PVDF materials, Kynar 740 and MG15, both from Arkema, as well as propionic acid (PA), lithium nitrate (LiNO_3_), activated carbon (AC), and aerosil 200 (AER) as additives in the dope solution. Dope solutions were prepared with 17 wt % PVDF and an additive concentration in the range of 1–5%. The authors observed that due to the use of different types of PVDF, i.e., polymers with characteristics that formed polymer solutions with different viscosities, it was possible to obtain membranes with different morphologies and transport properties, even when the polymer solutions were prepared using the same composition, temperature, and additives, as well as applying the same spinning conditions. Hollow fibers synthesized from Kynar740 and 5% of PA presented a higher CO_2_ flux than other combinations of polymer and additive. They attributed this to the final hollow fiber morphology, which is related to synthesis parameters, such as the dope solution, bore liquid, and precipitation bath concentrations, as well as mechanisms of interaction between the additive and the dope solution during precipitation. It is noticed that the synthesis of PVDF hollow fibers for membrane contactors, used in CO_2_ absorption, presents a number of challenges and possibilities. It is of great value to understand the effects of polymer solution composition, precipitation bath, bore liquid composition, and spinning parameters on the morphology, since it directly influences transport properties. Moreover, in the case of membrane contactors, the pore size on the surface is highly important, because the decrease of the mean pore diameter at the hollow fiber outer surface, which is in contact with absorbent liquid, prevents wetting.

Therefore, the focus of this paper was to produce and characterize a PVDF hollow fiber membrane using adipic acid (AD) as an additive in the dope solution. The PVDF hollow fibers produced were used in the gas–liquid membrane contactors process, aiming at CO_2_ capture. The main steps investigated in this work are shown in Figure 1. AD was selected as an additive based on the results of Pereira et al. with PA.^24^ The authors found that PA promoted a porous structure due to its capacity to form a Lewis acid–base complex with NMP. Therefore, the object of using AD is its ability to form acid–base Lewis complexes. AD is a dicarboxylic acid (Lewis acid), which has been previously proved to form complexes with NMP (Lewis base).^25^ The authors observed that, in addition to the acid hydrophobicity, the capacity of the additive (AD) to form complexes with the solvent (NMP), as well as the amount of solvent molecules that do not form complexes, will strongly affect the miscibility region. Fritzsche et al. used Lewis acid base complexes to obtain gas separation membranes.^26^ The authors suggest that it is possible to use a higher additive content in the presence of such complexes in the polymer solution. In the phase separation process, the immersion of the polymer solutions into a precipitation bath composed of a nonsolvent that presents a high dielectric constant promotes a fast complex dissociation, which leads to polymer precipitation. Due to the faster precipitation, compacting and conformational rearrangement of the polymer segments is inhibited, which can increase the free volume in the region of the separating layer. In this scenario, we selected AD as the additive in the dope solution and NMP as the solvent.

## Methodology

### Materials

PVDF Kynar 700 series, from Arkema, was dried at 60 °C in an oven for at least 24 h before dope solution preparation, and NMP, purchased from Vetec. AD, from Riedel-de Han, used as an additive in the dope solution, was also previously dried. NMP, distilled water, and polyvinylpyrrolidone (PVP) 360 kDa were used in the bore liquid. PVP was purchased from Sigma-Aldrich. Ethanol (95%) and hexane, both from Vetec, were applied for the membrane drying step in the exchange solvent procedure. CO_2_ 99.99%, nitrogen (N_2_) 99.99%, and ultrapure He, purchased from Linde Gases, were used, as received, for hollow fiber characterization. Microfiltered water, produced in-house, and NaCl (99%), from Vetec, were used to prepare the liquid absorbent solution, in performance tests. NaOH (98%), from Vetec, was used to maintain the constant pH of the liquid absorbent. The dope solution was prepared by a mechanical stirrer at room temperature (23 °C) until the polymer was completely dissolved. Dope was composed of PVDF (17 wt %), NMP (78 wt %), and AD (5 wt %). Bore liquid used was prepared by stirring 30% demineralized/microfiltered water and 70% NMP, as well as 10% PVP based on the total mass of water and NMP.

### Hollow Fiber Preparation

Hollow fiber membranes were produced by the NIPS process. This technique consists of spinning the dope solution into the precipitation bath, which was composed of microfiltered water, as a nonsolvent, in the present work. The immersion of the polymer solution into a nonsolvent precipitation bath induces phase separation. The spinneret used had two concentric orifices, where the outer orifice was used to flow dope solution and the inner orifice was used to flow bore liquid, which creates a hollow fiber lumen. During preparation, the flow of the dope solution and bore liquid was kept at 8.4 g/min and 2.3 mL/min, respectively.

### Module Assembly

In order to test the performance of PVDF hollow fibers, poly(vinyl chloride) (PVC) modules containing the PVDF hollow fibers were prepared. A bundle of hollow fibers were introduced into the module, and the sides of the module were sealed with a two-component epoxy mixture to separate the gas and liquid streams during the CO_2_ removal process.

**Figure 2:**
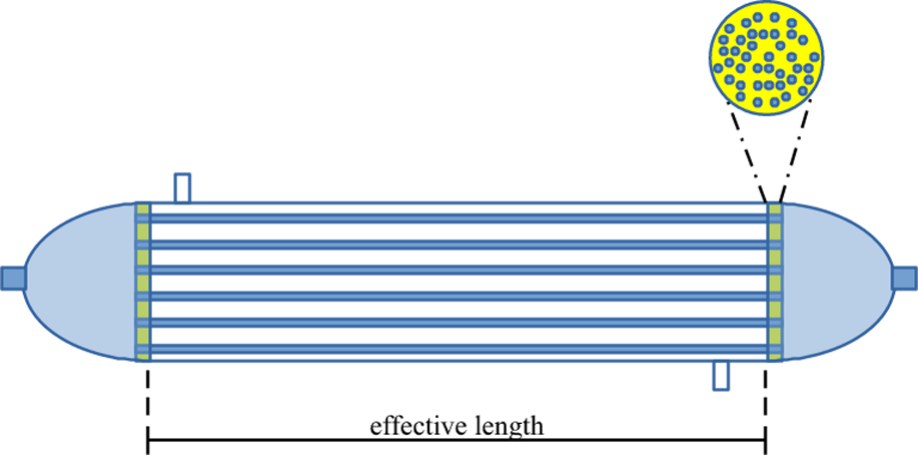
Schematic representation of in-house module II with 40 PVDF hollow fibers randomly packed.

Two types of modules were manufactured with an effective length of 15 cm, containing a bundle of ten fibers (Module I) and another bundle of 40 fibers (Module II), respectively. A schematic representation of the in-house module is shown in Figure 2. In addition, the dimensions and characteristics of the modules are shown in Table 1.

**Table 1:** In-House Module Specification

parameters	module I	module II
Length, *L* (cm)	15	15
Number of fibers, *n*	10	40
Shell diameter, SD (cm)	1.27	2.54

## Characterization

### Scanning Electron Microscopy

Hollow fiber morphology was evaluated by SEM. The samples were fractured by cryogenic technique after immersion in liquid nitrogen in order to not deform while breaking them. Then, the samples were fixed on stubs and covered with gold by cold sputtering using a Sputter Q150R ES from Quorum Technologies. After metallization, SEM images were obtained using an FEI Quanta 200 microscope applying an acceleration voltage of 20 kV.

### Helium Ion Microscopy

Helium ion microscopy (HIM) was performed to clearly observe the membranes, and it had successfully been used for membrane characterization by Imbrogno et al.^27^ It is a type of microscopy that uses a beam of helium ions to obtain images with a resolution around 1 nm instead of using an electron beam. Due to the beam used, there is no need to metalize the sample. Therefore, HIM is applied to obtain high-resolution images of insulating materials without losing information due to the gold coating. Hollow fiber samples were displaced on carbon tape over an aluminum support. In order to visualize the membrane cross section, a cryogenic fracture procedure was applied, as described for SEM analysis. HIM samples were analyzed by an Orion Nanofab from Zeiss, under 30 kV and 0.5 pA.

### Gas Permeation Tests

**Figure 3:**
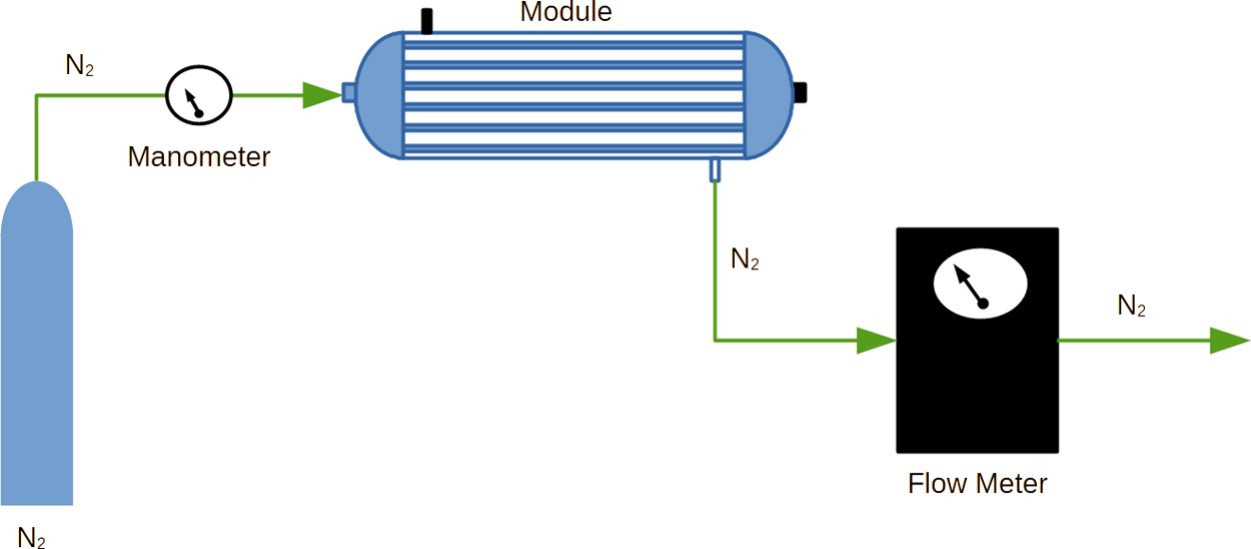
Gas permeation experimental apparatus.

Gas permeance was measured by flowing pure nitrogen (N_2_) through membrane modules, at 1 bar, as depicted in Figure 3.
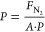
1

Permeance was calculated by eq 1, where *P* is the permeance (cm^3^/(cm^2^ cmHg s)), *F*_N_2__ is the N_2_ flow (cm^3^/s), *P* is the feed gas pressure (cm Hg), and *A* is the external surface area of the hollow fibers (cm^2^), inside the module.

### Porosity

Porosity (ϵ) was calculated by the ratio between the void volume and geometric volume (*V*_t_) of the hollow fiber wall, considering it integrally dense. The void volume was obtained by the difference between the geometric volume (*V*_t_) and the real volume (*V*_r_). Therefore, porosity (ϵ)) can be calculated in a simplified way, as presented in eq 2.
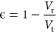
2

Geometric volume (*V*_t_) can be calculated by eq 3, and it represents the volume of the solid cylinder with length *L*, whose wall is formed by a circular ring with an inner diameter (*d*_i_) and an outer diameter (*d*_e_). Real volume (*V*_r_) was obtained by helium pycnometry using AccuPyc 1330 equipment from Micromeritics.
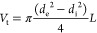
3

### Performance Tests

**Figure 4:**
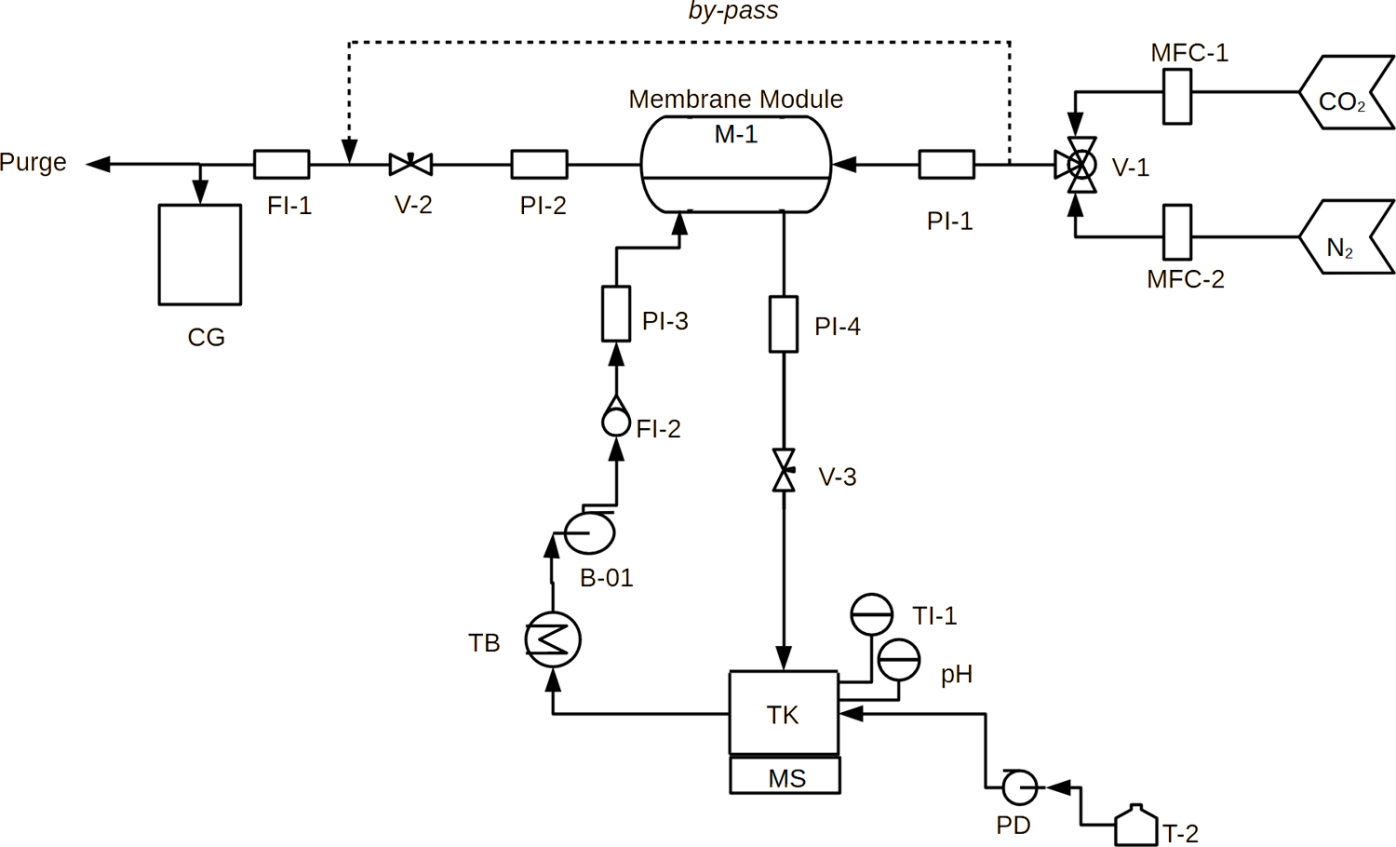
Schematic diagram of the membrane contactor process.

Performance tests of PVDF hollow fibers were carried out using the experimental apparatus depicted in Figure 4.

The apparatus consists of a module (M-1), an acrylic tank (T-1), a tank for the NaOH solution (T-2), and a computer (IC-1) to adjust the gas flow meters and equipment, instruments, and valves listed below:

1. Equipment
2. Gas chromatograph, MicroCG CP 4900, Varian
3. Thermostatic bath, Polystat, Cole-Parmer
4. Pump to flow absorber liquid, Gear Pump Drive, Cole-Parmer gear pump
5. Magnetic stirring plate, Framo, Gerätetechnik
6. Metering pump, Titronic, Schott
7. Instruments
8. Mass flow controller for CO_2_, 100 N mL/min, Brooks Instrument
9. Mass flow controller for N_2_, 100 N mL/min, Brooks Instrument
10. Process flow current flow meter, bubble flow meter
11. Liquid flow meter, KDS-120, Heinrichs Messtchinik
12. pH meter, pH meter DM-22, Digimed
13. Temperature sensor, Digimed
14. Inlet gas pressure gauge, manometer 0–2 bar, Famabras
15. Exhaust gas flow pressure gauge, manometer 0–4 bar, Famabras
16. Inlet liquid pressure gauge, manometer 0–2 bar, Famabras
17. Output liquid pressure gauge, manometer 0–4 bar, Famabras
18. Valves
19. Valve for mixing pure gases, three-way valve, Swagelok
20. Valve for adjusting the pressure of gas stream, needle valve, Hooke
21. Valve for adjusting the pressure of liquid stream, micrometer valve, Swagelok.

The liquid absorbent was added to the acrylic tank and pumped in a closed loop through the membrane module and then back to the acrylic tank. The liquid absorbent temperature was controlled by a thermostatic bath. The liquid pH was adjusted with NaOH solution, which was added to the tank using a metering pump. The gas mixture was produced inline by controlling CO_2_ and N_2_ flows using mass flow controllers. During the adjustment of the liquid temperature, the gas mixture flow was directed to purge using a bypass. When the temperature of the liquid became constant and equal to the proposed temperature for the experiment, the gas stream was redirected to pass through the membrane module. The pressures of the gas and liquid streams were gradually increased to achieve the experimental operating pressure. During this procedure, the liquid pressure was kept 0.2 bar higher than the gas pressure, avoiding wetting of the membrane.^11,16,17,28−31^ The gas mixture composition and flow rate were analyzed by a gas chromatograph and bubble flow meter, respectively. The flux of a component was calculated by eq 4.
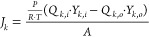
4where *J*_k_ is the flux of component *k*, *P* is the pressure of the gas stream, *T* is the temperature of the gas stream, *R* is the universal gas constant, *Q_k_* is the flow rate of component *k*, and *Y_k_* is the volumetric fraction of component *k*, in the gas stream. The subscript *i* means input from the module, whereas subscript *o* means output from the module. The percentage removal of a component was calculated by eq 5.
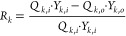
5where *R_k_*, *Q_k_*, and *Y_k_* are the percentage removal, flow rate, and volumetric fraction of component *k* in the gas stream, respectively.

The gas phase consisted of a mixture of 10% CO_2_ and 90% N_2_ under a pressure of 2 bar with a flow rate of 100 mL/min. In the liquid phase, an NaCl solution of 3.5% was used under a pressure of 2.2 bar and a flow rate of 50 L/h. The liquid phase temperature was maintained at 10 °C, and the pH was kept at 8.

## Results and Discussion

During spinning, it was possible to observe that when an air gap distance of 4 cm was used, the polymer solution extrusion was not able to form stable hollow fibers. This probably occurred due to the low velocity of precipitation before immersion into the precipitation bath. Light transmission tests carried out in a previous work, with the same polymer solution, indicated that it took more than 100 s for the polymer solution to start precipitation. In the light transmission tests, the polymer solution was cast on a glass plate, resulting in flat sheet membranes. The precipitation bath was water, as used in the external precipitation bath during spinning. During spinning, it is also important to notice that the bore liquid was composed of a water/NMP mixture, which also promotes a precipitation delay when compared with pure water. Therefore, it is acceptable that the use of a 4 cm air gap did not help form hollow fiber membranes since precipitation from the outer and inner layers did not favor precipitation. Hence, the following tests were then performed with membranes obtained by using the lowest air gap distance, which favors a faster precipitation, i.e., immersion into the precipitation bath immediately after polymer solution extrusion), so that the polymer solution instantly contacts water, using an air gap distance of approximately null.

The morphology of PVDF membranes was characterized by SEM and HIM. For comparison, the morphology of commercial membranes was also evaluated by SEM analysis.

**Figure 5:**
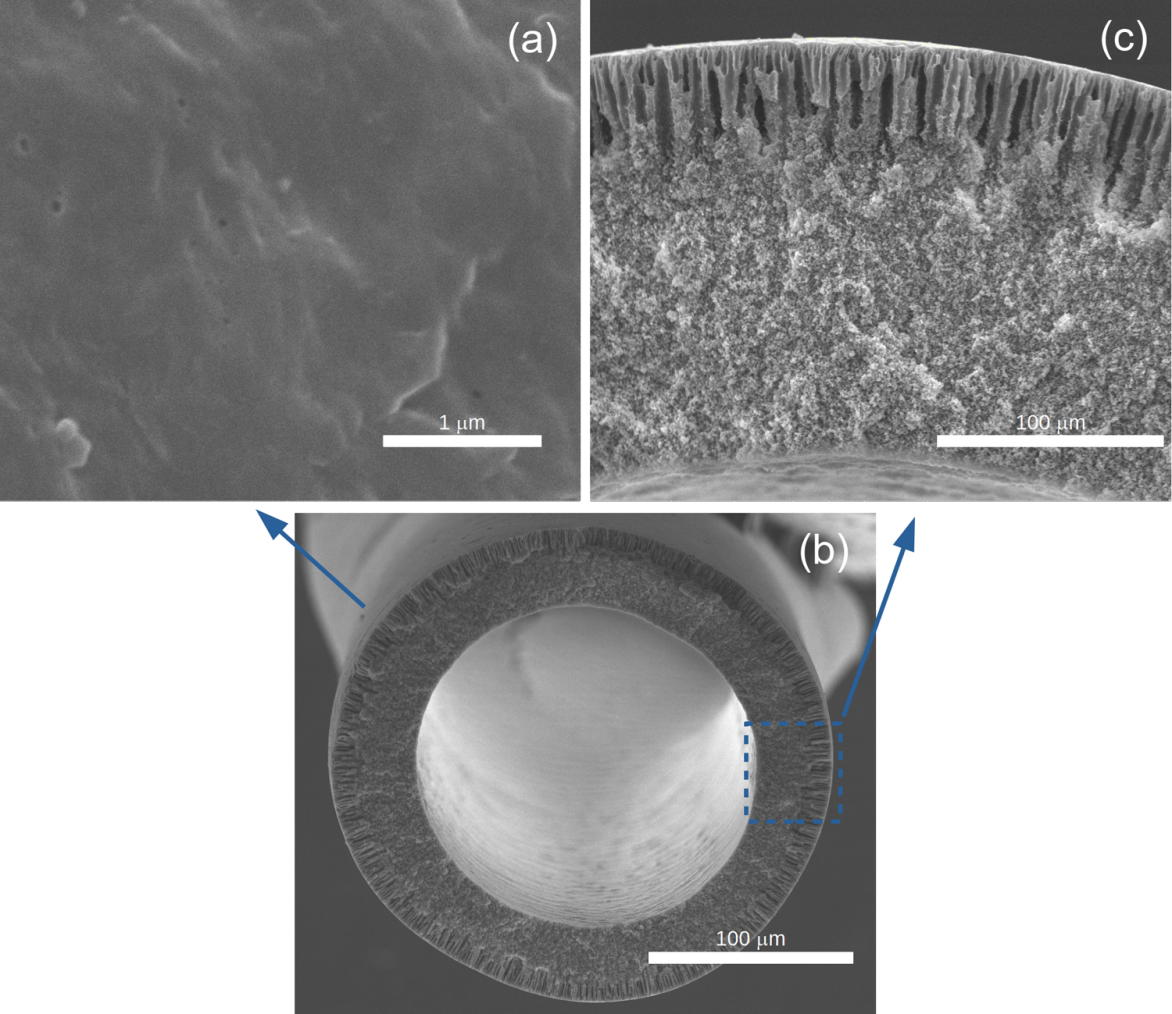
SEM photomicrograph of PVDF hollow fiber: (a) outer surface; (b) cross Section; (c) cross section details.

SEM analysis, shown in Figure 5, indicates that PVDF hollow fibers had outer and inner diameters of 1126 and 836 μm, respectively. From the photomicrographs, an anisotropic morphology was also observed. There are different porous regions in the cross section of the membrane. In the inner region, the pores are interconnected and exhibit a sponge-like morphology. Near the outer surface, the presence of macropores is evident, where the pores have a finger-like morphology.

The mean pore diameter, measured through a combination of SEM and HIM, is approximately 30 nm. Additionally, the membranes have a porosity of 63%, as calculated by helium pycnometry. These properties indicate that the PVDF hollow fibers possess a well-defined pore structure and high porosity, which are advantageous for applications requiring efficient gas and liquid transport such as in membrane contactors.

Macropore formation is expected when a fast demixing occurs at the interface between the polymer solution and the precipitation bath, which increases the polymer concentration in this region. This effect may create a resistance to mass transfer between the polymer and the precipitation bath, allowing the growth of nuclei in the sublayer. These conditions for macropore formation were provided since a fast demixing is expected when water from the precipitation bath comes in contact with the polymer solution.

Additionally, the presence of AD may also provide fast demixing in this region. AD is a dicarboxylic acid that forms a complex with NMP.^24^ Hence, the use of AD as an additive provides a polymer solution composed of a Lewis acid–base complex (AD – Lewis acid and NMP – Lewis base). It is expected that, after immersion into water, a fast complex dissociation may be achieved due to the high dielectric constant of water, and consequently, the polymer solution receives a high amount of nonsolvent, which promotes precipitation.

A fast precipitation near the outer surface and a stable sublayer promote nuclei growth from the outer layer to the inner layer, leading to a high incidence of macropores. On the other hand, the use of AD may also increase the polymer solution viscosity, which further favors the formation of a mass transfer resistance between the precipitation bath and the sublayer. Furthermore, the presence of solvent in the bore liquid also favors the stability of the sublayer near the inner surface for a longer period; therefore, it also promotes macropore growth from the outer to the inner surface.

**Figure 6:**
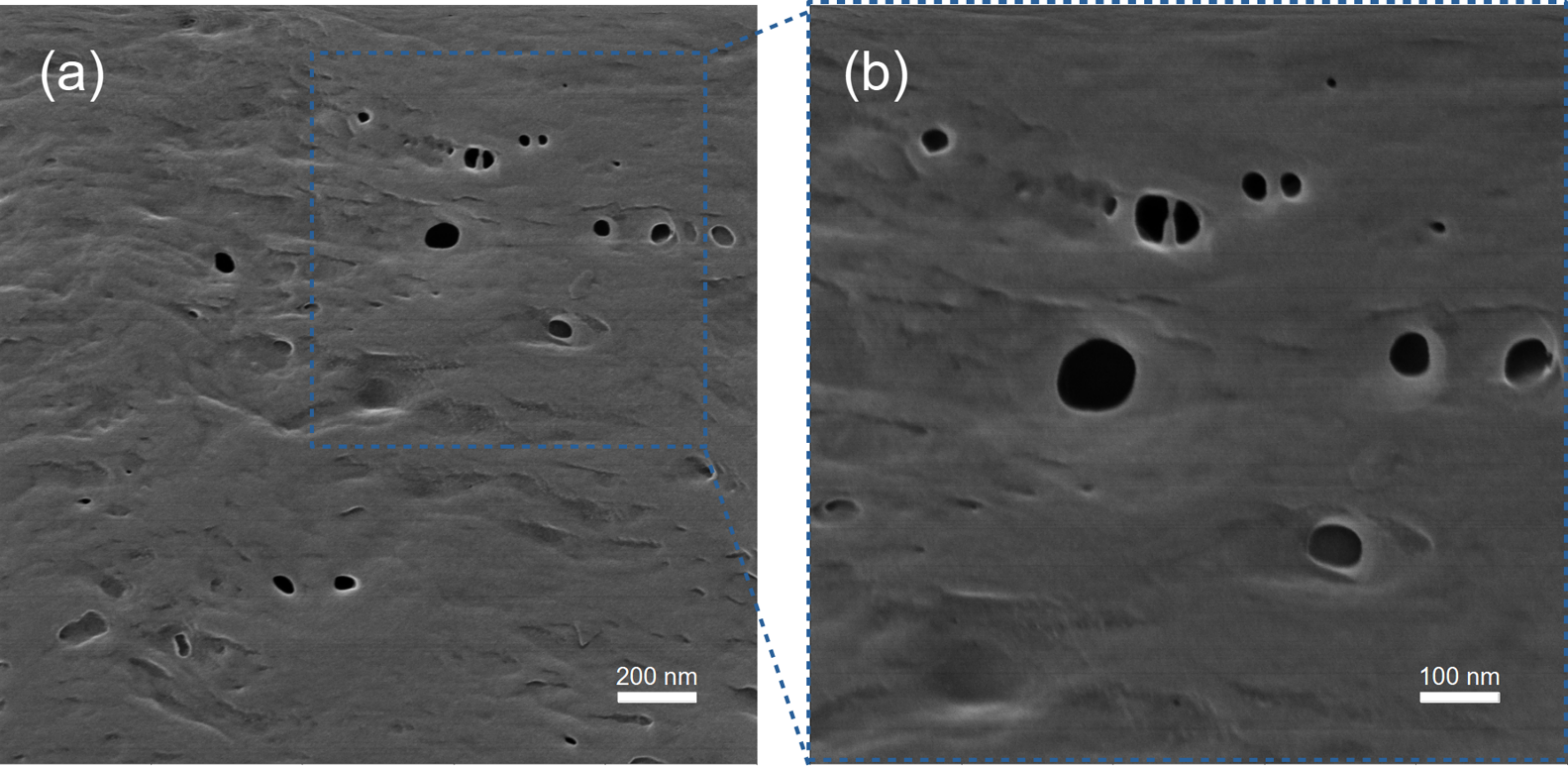
HIM analysis of the PVDF hollow fiber outer surface: scale: (a) 200 nm and (b) 100 nm.

**Figure 7:**
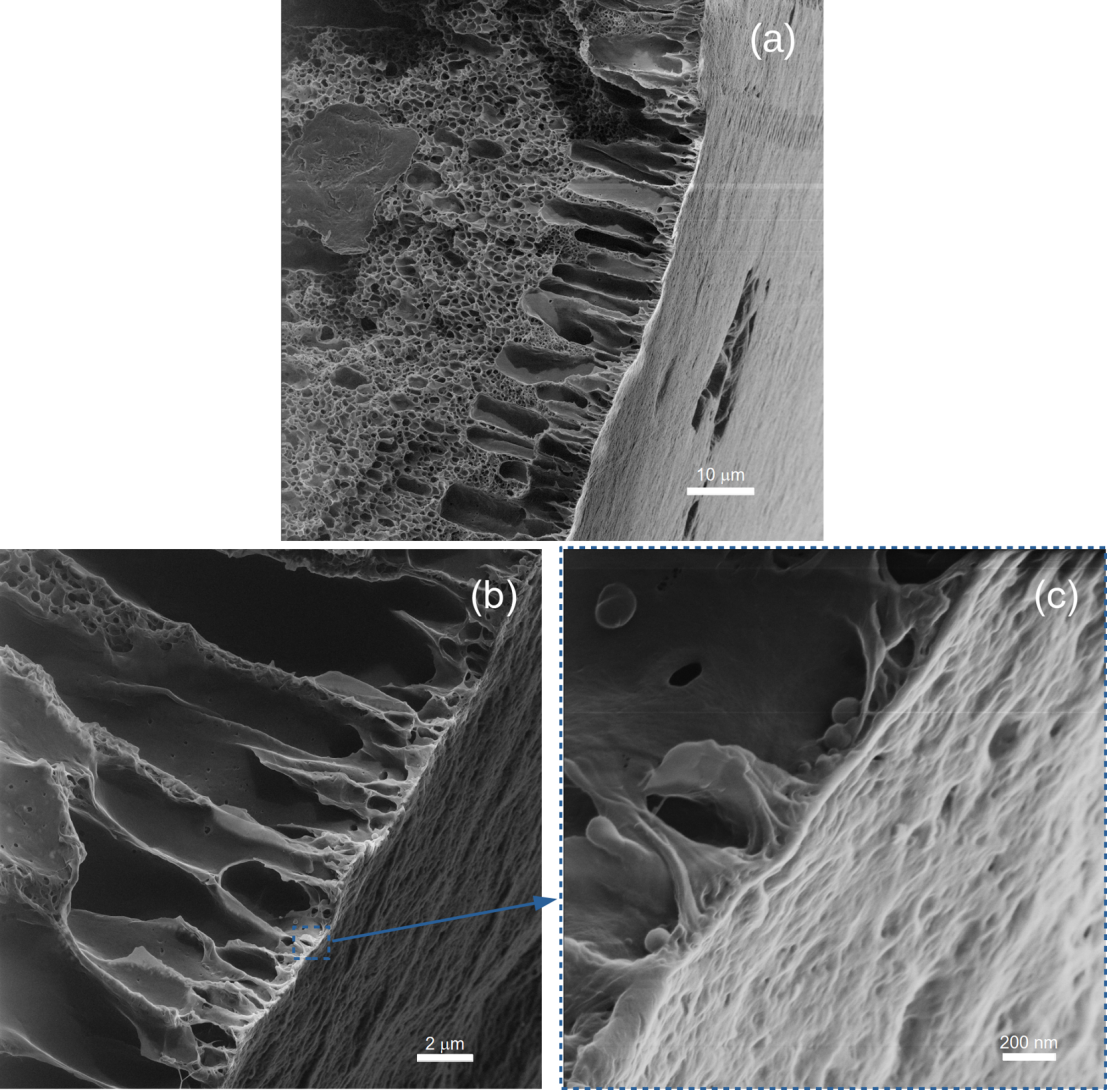
HIM analysis of PVDF hollow fiber showing cross section details: scale (a) 10 μm, (b) 2 μm, and (c) 200 nm.

The morphology of PVDF hollow fibers was also characterized by HIM. Figure 6 clearly shows that, in the outer surface, pores are on the nanometric scale. The pore diameters are in the range of 10–100 nm, with most of them in the range of 10–40 nm. It can also be observed that the surface nanopores are connected to the inner layer in the region of macropores, which facilitates permeation through the cross section, as depicted in Figure 7.

HIM analysis is in agreement with SEM results; however, the HIM technique allowed for a clearer observation of the outer surface and the finger-like porous region, which grows from the outer to the inner layer. The presence of small pores at the membrane surface may favor process efficiency, as it may prevent membrane wetting and, consequently, its nondesirable effects.^15^ El-Naas et al.^28^ observed that the presence of large pores may enable membrane wetting, even under nonwettable conditions, such as when using a hydrophobic polymer such as PP, in cases of high solvent and low gas flow rates. Therefore, the nanopores at the surface of the produced PVDF membranes may contribute to the success of the contactor process performance.

PVDF hollow fibers were characterized by helium pycnometry in order to obtain membrane porosity. The tests resulted in an average value of 63%. This result is in agreement with the morphology observed by SEM and HIM techniques, confirming that it is more porous than the PP commercial membrane. Table 1 summarizes all of the characteristics of the synthesized PVDF hollow fiber.

Ghasem et al.^22^ observed that the increase in PVDF concentration reduced membrane porosity. In their work, the porosity varied from 45, 39, 36, 34, and 32% to 25, 28, 30, 32, and 34%, respectively. PVDF membranes were obtained by Fosi-Kofal et al.^32^ using LiCl as an additive, in the composition PVDF/LiCl (18%/3%) and the addition of CaCO_3_ from 0 to 30%. The porosity varied from 78 to 81%. According to the authors, the presence of CaCO_3_ increased the porosity due to the formation of macropores. The use of AD in the present work led to porosity values in agreement with the values reported in the literature, which suggests that AD is a promising candidate for preparing porous membranes.

PVDF hollow fiber membranes presented a nitrogen permeance of 7.15 × 10^–3^ cm^3^/cm^2^ s cmHg (7.15 × 10^3^ GPU) at 1 bar, which is nearly 1 order of magnitude higher than that of the commercial PP membrane (7.09 × 10^–4^ cm^3^/cm^2^ s cmHg).

In our previous work,^33^ flat sheet membranes were prepared using the same dope polymer solution and water as the precipitation bath. The authors found a significantly higher nitrogen permeance, 1.5 × 10^5^ GPU. However, it is important to emphasize that in the case of flat sheet membranes, the polymer solution is cast on a glass plate, which acts as an inert surface. Precipitation occurs only due to immersion into the external precipitation bath; therefore, it takes longer for the sublayer to precipitate, which favors the formation of larger pores.

During hollow fiber membrane preparation, i.e., during spinning, in addition to the external precipitation bath, a bore liquid is used as an internal precipitation bath. Therefore, a hollow fiber cross section is formed due to the effect of immersion into two precipitation baths – from the outer and inner layers – which promotes a faster precipitation that consequently reduces the pore size closer to that of the inner layer.

The resulting morphology of the flat sheet membrane provides less mass transport resistance when compared to that of the hollow fiber, leading to higher gas permeation. On the other hand, the hollow fiber morphology may contribute to avoiding membrane wetting.

The results of nitrogen permeance of the PVDF hollow fibers are consistent with the porosity values previously measured by helium pycnometry and with SEM and HIM analyses. Naim et al.^23^ investigated the effect of lithium chloride as an additive for PVDF solutions. When the LiCl concentrations were 3 and 5 wt %, the authors found nitrogen permeance values of 8.25 × 10^–3^ and 1.0 × 10^–3^ cm^3^/cm^2^ s cmHg, respectively. According to the authors, the mean pore radii calculated using the nitrogen permeance were 40 and 28 nm for membranes obtained when LiCl was added to the polymer solution at concentrations of 3 and 5 wt %, respectively. These results (nitrogen permeance and pore diameter) are in agreement with those of the present work. These findings indicate that the PVDF hollow membranes obtained in this work are promising for contactor applications.

Two different modules were fabricated for use in gas–liquid membrane contactors using PVDF hollow fibers. The PP commercial module presented a satisfactory result when comparing the module CO_2_ removal to both PVDF modules. CO_2_ removal (%) values were 4.95, 18.49, and 69.10 for modules I, II, and III, respectively, i.e., CO_2_ removal in the PP module was four times higher than that in PVDF module II and 14 times higher than that in PVDF module I. These results are expected, since the gas residence time in the PP module is much higher than that in the PVDF modules, as well as the membrane area.

However, to precisely compare CO_2_ removal by modules with different characteristics, it is necessary to normalize it by taking into account the number of fibers and their length. CO_2_ removal values (% m/fiber) were 3.31, 3.08, and 0.31 for modules I, II, and III, respectively. In this comparison, PVDF modules I and II present almost the same CO_2_ removal value, whereas the PP module presents a much lower value, approximately 10 times lower. These results indicate the satisfactory performance of PVDF hollow fiber membranes.

It is important to notice that PVDF module II was not able to operate at a higher flow rate (50 L/h) due to a pressure drop in the absorbent liquid stream. Therefore, the experiments for PVDF module II were carried out by using a lower flow rate (30 L/h). Nevertheless, PVDF module I presented a CO_2_ flux of 118 × 10^–6^ mol/m^2^ s, whereas PVDF module II had a CO_2_ flux of 111 × 10^–6^ mol/m^2^ s, i.e., the flux values were quite similar. On the other hand, the PP commercial module had a flux of 47 × 10^–6^ mol/m^2^ s, which is 2.5 times lower than those of the PVDF modules.

However, it is important to emphasize that the modules have different dimensions and numbers of fibers. These variables directly influence the hydrodynamics in the shell module. Mass transfer in a membrane contactor can be modeled using a three-resistance model: one in the gas phase, one in the membrane, and one in the liquid phase. Considering that the membrane is not wetted, the major resistance to transport occurs in the liquid boundary layer. These correlations show that the Reynolds number directly influences this resistance.^5,7,8,15,29^ Hence, the Reynolds number (*Re*) for each module is suitable for comparison. According to the *Re* number, all of them had laminar flow, although they were very different from each other: *Re* numbers were 524, 106, and 19 for PVDF modules I, II, and PP module III, respectively. Even though this difference exists, the results from PVDF module II indicate that the PVDF hollow fiber obtained by using AD as an additive is promising for use in gas–liquid contactor processes.

## Conclusions

PVDF hollow fibers, prepared using AD as an additive in polymer dope, showed an anisotropic structure containing a sponge-like region inside the hollow fiber and an outer region containing finger-like macrovoids. HIM clearly showed that the outer surface of the PVDF hollow fiber has nanometric pores in the range of 10–40 nm. The presence of nanopores at the outer surface of hollow fibers is satisfactory since it may prevent a decrease in process performance due to membrane wetting, as reported in the literature. When normalizing CO_2_ removal due to the modules with different characteristics, by taking into account the number of fibers and length, it could be seen that PVDF hollow fibers had better performance CO_2_ flux than commercial PP hollow fibers. PVDF hollow fibers seem to be adequate to be applied in CO_2_ removal using a membrane contactor due to their morphology and hydrophobicity.
